# The iPhone Measure app level function as a measuring device for the weight bearing lunge test in adults: a reliability study

**DOI:** 10.1186/s13047-019-0347-9

**Published:** 2019-07-09

**Authors:** Helen A. Banwell, Hayley Uden, Nicole Marshall, Carlie Altmann, Cylie M. Williams

**Affiliations:** 1 International Centre for Allied Health Evidence (iCAHE), University of South Australia, Adelaide, South Australia 5001 Australia; 2 School of Health Sciences, University of South Australia, Adelaide, South Australia 5001 Australia; 3 Allied Health, Peninsula Health, Frankston, Victoria 3199 Australia; 4 School of Primary and Allied Health, Monash University, Frankston, Victoria 3199 Australia

## Abstract

**Background:**

Ankle joint range of motion is a frequently assessed measure used by health care clinicians who manage lower limb pathologies to identify ankle equinus and/or other joint motion concerns that may negatively impact on function. The purpose of this study was to assess a new iPhone application (the level function of the ‘Measure application’), for measuring the weightbearing ankle lunge test in a healthy adult population (reliability) and measuring known angles (validity) when compared to a digital inclinometer.

**Methods:**

To determine intra-rater reliability, inter-rater reliability and concurrent validity, 168 measures were conducted on 21 participants. Participants were preconditioned prior to assessment, and two experienced raters measured ankle dorsiflexion range of motion in the knee extended and knee flexed positions of the weight bearing lunge test, using an iPhone level function (of the Measure application) and a digital inclinometer in a randomised order, over two timepoints. Concurrent validity was also determined by comparison of measures of the two devices at known surface angles (0 and 15 degrees) in multiple planes. Reliability and validity were determined with intraclass correlation coefficients, concurrent validity was explored with the Bland Altman plot and an intraclass correlation coefficient. The Standard Error of the Mean and the minimal detectable change were also explored.

**Results:**

The intra-rater reliability using the iPhone and inter-rater reliability using the digital inclinometer, in the knee extended position, were ICC 0.85 respectively, indicating good reliability. All other intra-rater reliability and inter-rater reliability for both devices and both leg positions were over ICC 0.90, indicating excellent reliability. Concurrent validity between the two devices on a flat and known angle surface were ICC 1.0 (Limits of Agreement − 1.0 to 0.61), indicating excellent validity, with good validity demonstrated by a Bland Altman plot of all measures in all positions (ICC of 0.84 (Limits of agreement = − 4.51 to 6.49)).

**Conclusion:**

The use of the iPhone level measure, within the Measurement App has demonstrated to be an easy and reliable measurement tool to determine ankle joint dorsiflexion during the weightbearing lunge test in healthy adults.

## Background

A reduced range of ankle joint motion (i.e. ankle equinus) has been shown to have a negative impact on lower limb function and economy of gait in healthy and pathological populations [1–6]. Clinicians involved in the assessment, diagnosis and management of foot and leg conditions often identify restrictions of ankle joint motion and prescribe interventions, such as stretch and strengthening programs, with re-assessment of measures used to determine success [1, 5]. This requires the measure used to be repeatable and consistent. The identification of reduced ankle joint motion can be measured clinically via weightbearing and/or non-weightbearing methods, with the weight bearing lunge test deemed the preferred method due to improved capture of full joint excursion [7–10].

In clinical practice digital inclinometers are a frequently used measuring tool, which have proven to be reliable and valid for the weight bearing lunge test [8, 11] and are comparable to two-dimensional motion capture systems [12]. However, digital inclinometers may be considered costly for the average clinician and are not often accessible by clients/carers who may wish to assess range of motion changes at home. With the advances in technology, some applications (Apps) have been reported as suitable substitutes. Specifically, the Tiltmeter App and the iHandy App (*available on smart phones/tablets*) have been shown as reliable measures of ankle joint dorsiflexion [13, 14]. These have the additional benefits of being cheap, easily accessible and quick to administer [13]. Unfortunately, with rapidly changing technology, these Apps become outdated and unsupported, as demonstrated with the recent discontinuation of the Tiltmeter App for iPhone users. With Apple’s recent software upgrade (operating systems IOS 7 and above) a new Measure App which includes a ‘level’ function has been introduced. This level function, if reliable, would potentially be a suitable alternative to the discontinued Tiltmeter App with the additional bonus of being included in the Apple App suite (that is, it is standardly installed/upgraded with each software upgrade). Furthermore, in Australia, iPhone users account for 45% of the smart phone market share (8.6 million users) [15], meaning the Measure App is freely accessible to a large population of smartphone users. To be confident in its use in the clinical setting, however, determination of the psychometric properties is required.

The primary aim of this study was to determine the intra and inter-rater reliability of the level function of the Measure App compared to a digital inclinometer. The secondary aim was to determine the concurrent validity of the two tools (i.e. how well does the level function measure when compared to the digital inclinometer).

## Methods

The study design was to determine intra-rater and inter-rater reliability of the weight bearing lunge test with both the knee extended and knee flexed, using the digital inclinometer and the iPhone Measure App. The study was also designed to determine the concurrent validity between the two tools.

### Raters

Two podiatrists (CA and NM) conducted all measurements. Both raters (CA and NM) had 8 years clinical experience, have post-graduate research training and use the WBL measurement technique routinely during clinical practice. Raters were involved in the development of the protocol, reviewed the final protocol and practiced the measure on two participants (not included in the final study) 1 week prior to conducting the study to allow open discussion regarding procedure.

### Participants

A convenience sample of 21 participants were recruited from the University of South Australia podiatry student cohort. Students were alerted to the study by email correspondence outlining the study aims and disseminating participant information sheets and consent forms for participants to consider in their own time. To minimise the risk of coercion, all correspondence informed students that involvement in the study was voluntary and could be withdrawn at any time, and participants indicated their willingness to be involved by returning a signed consent form to an administrator external to the podiatry course. Participants were excluded from the study if they had: foot pain or injury within the past 6 months; any past foot or ankle surgery; or a neurological or inflammatory condition affecting gait. Ethics approval was obtained from the University of South Australia’s Human Research Ethics Committee (Approval number 201357).

### Procedure

Two tools were compared within this study. The Geo Fennel S-Digit Mini Inclinometer (digital inclinometer), (GSR Laser Tools, Perth, Australia); and the level function available via the Measure App, a free App available on the iPhone smart phone (operating systems IOS 7 and above). For this study the iPhone 6S was used (Apple Inc., Cupertino, CA, USA). Prior to testing, the digital inclinometer and iPhone Measure measures were compared for consistency on identical hard static flat and angled surfaces in multiple planes across three trials per angle. Prior to testing, the digital inclinometer was calibrated in accordance to industry requirements (Laser-Liner, UK), the iPhone was calibrated to zero degrees by placing it with its long axis on the floor.

**Fig. 1:**
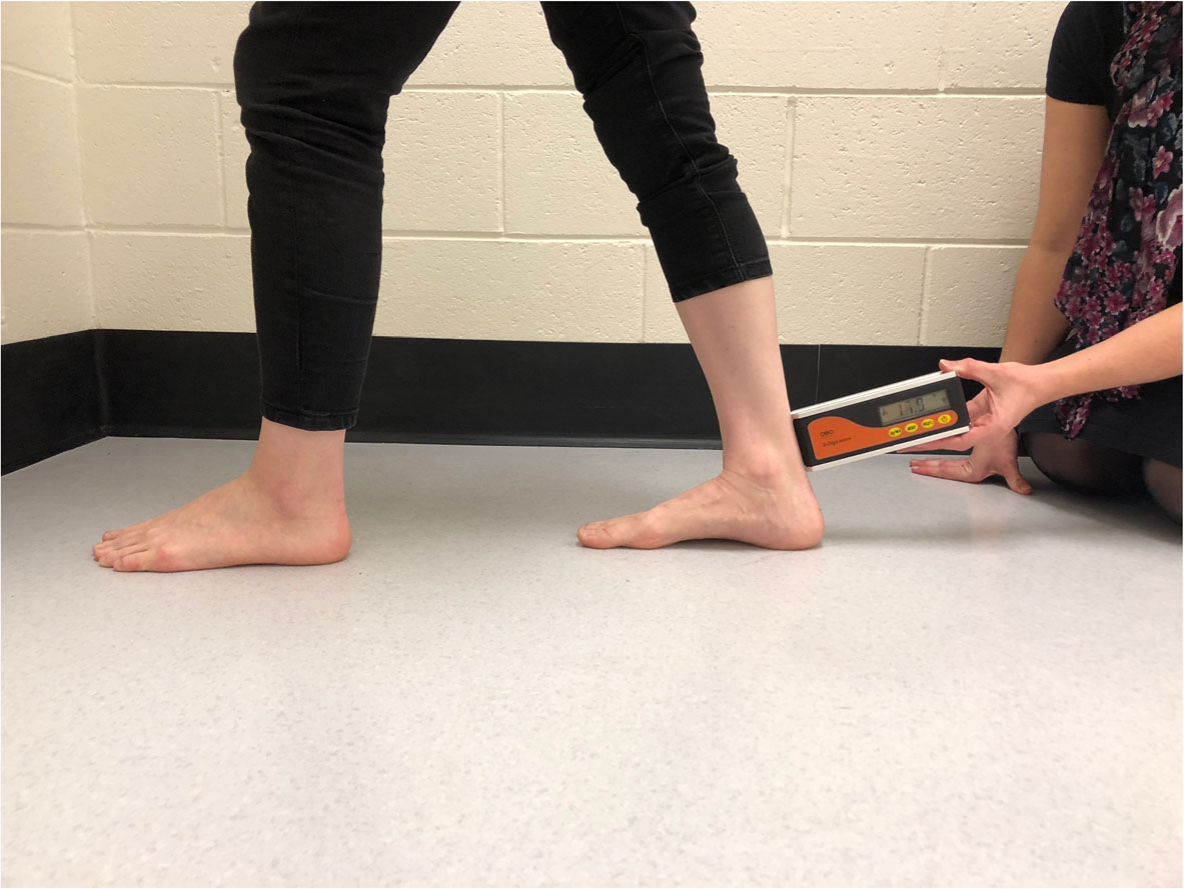
Weightbearing lunge test – knee extended position

**Fig. 2:**
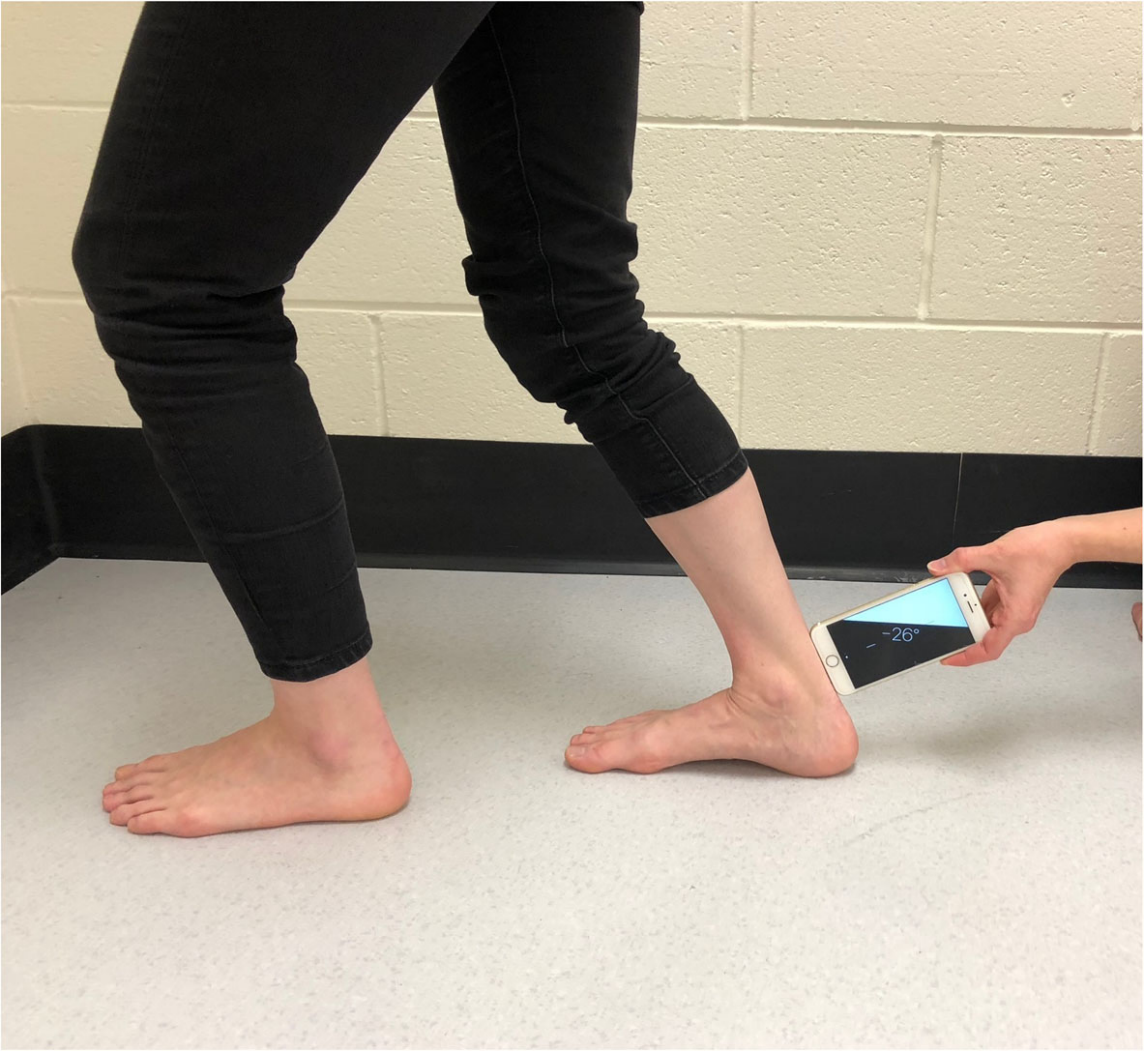
Weightbearing lunge test – knee flexed position

Participants were introduced to the study as a group and the WBL technique was explained and demonstrated. Prior to testing, each participant was required to hold a static WBL test stance in the knee flexed and knee extended position for 30 s each, three times. This preconditioning technique was chosen to allow participants to adopt the position easily. The WBL test protocol used during testing was consistent with Bennell et al. [16] as follows:

- Participants stood with their hands shoulder width apart against the wall in front of them.
- The participants right leg was placed as far back as comfortably possible behind them whilst keeping their right heel to the ground, parallel to the left leg and perpendicular to the wall
- The rater assisted the participant to move their right foot back until the lunge position could be held whilst the heel remained on the floor and the knee aligned over the second toe [16]
- WBL measures were then taken with the knee extended (Fig. 1) and the knee flexed (Fig. 2).
- A single measure was taken at each time point, in each position by each of the raters.

To measure the WBL, the short arm of the device was placed flat against the posterior heel, approximately one-centimetre superior to the posterior calcaneal tuberosity and held perpendicular to the shank of the tibia until the measure (in degrees) remained fixed (Figs. 1 and 2). The degree was determined by the long axis of the device relative to the horizontal (zero degrees). This is consistent with the method of measurement and position of measuring devices in similar studies [13, 17].

Testing occurred over one four-hour session. The order of participants and the measuring device used were randomised by computer table [18] and administered independently to the raters (HB). Measures were collected for the right foot only to satisfy the assumption of data independence [19]. To minimise recall, participants were measured behind a partition that allowed the practitioner to visualise the person from their knees down only. The author group considered the sample size large enough to ensure raters were unable to remember the result; and the time space between retesting participants (minimum of 30 min) was appropriate to not cause fatigue to the target muscle group.

### Data analysis

Participant data were described in means (SD) and frequencies (%). The raw data from each rater, at the two timepoints, and each measured position, were normally distributed. Systematic error between timepoints were explored with t-tests. Significant differences between timepoints were considered where *p* < 0.05. The intra-rater reliability between timepoints for equipment was determined using the raw data with the intraclass correlation coefficient (ICC) (Model 3,1), 95% confidence interval (95% CI), Standard Error of the Mean (SEM) and the minimal detectable change (MDC). The SEM provided a measure of the variability and its calculation assisted in determining the MDC. The SEM was calculated with the raw data with the following formula: SEM = SD√(1-r) where r was the ICC for intra-rater reliability and SD was of the SD of measurement [20]. The MDC was calculated as the magnitude of change necessary in order to provide confidence that the change is not a result of random measurement error. The MDC was calculated as MDC = 1.96 x SEM x √2 [20]. The interrater reliability determined with all raw data collected from two raters, for each position and each measurement tool using ICCs (Model 2,2) 95% CI’s and Standard Error of the Mean (SEM). Concurrent validity was explored with the ICC and Bland Altman plot between the devices in both leg positions. The Bland Altman plot was used as a graphical display of agreement between measurement. It was used to assess the degree of agreement between the tools in all positions and by both raters, across the two timepoints. It also helps to identify the presence of bias. The Bland Altman was also used to calculate the mean difference between measures, the limits of agreement and the 95% confidence interval for the limits of agreement [21].

A minimum sample size of 18 was calculated to provide 80% power of detecting a ICC of 0.6 with a two-tailed alpha = 0.05 for the intra-rater reliability analysis [22]. The following ranges were used to report ICC data: < 0.5 = poor reliability, 0.5 to 0.75 = moderate reliability, 0.76 to 0.9 = good reliability, and > 0.90 = excellent reliability [22]. All data were analysed with Stata 15 [23].

## Results

Twenty-one participants met the eligibility criteria, gave informed consent to be part of the study and recorded their age, weight (kg) and height (cm), (Table 1). One hundred and sixty-eight measures were recorded.

**Table 1:** Participant characteristics

Characteristic	Mean (SD)	Range
Gender Male: Female		13:9
Age (years)	22.9 (1.4)	21 to 26
Height (cm)	173.7 (9.9)	152 to 189
Weight (kgs)	73.7 (10.9)	48 to 90

There were no differences between measures at each time point (*p* > 0.05). The intra-rater reliability for the tools were calculated for both raters (Table 2). The intra-rater reliability of the digital inclinometer was excellent for both leg positions (ICC = 0.91 to 0.97). The level function had good to excellent intra-rater reliability (ICC = 0.85 to 0.95), the lesser was with the knee extended (Table 2). There was also good to excellent inter-rater reliability between the raters with each tool. The digital inclinometer inter-rater reliability was good to excellent (ICC = 0.85 to 0.96). The level function had excellent inter-rater reliability between raters (ICC = 0.94 to 0.98). The lesser of both ICC scores was in relationship to the knee being in the extended position (Table 3).

**Table 2:** Outcomes of intra-rater reliability of the iPhone level function and the digital inclinometer for the weightbearing lunge test

Measure	Rater	Weight bearing lunge position	Mean (SD)	ICC	95% confidence interval	SEM	MDC
Intra-rater reliability of digital inclinometer	Rater 1	Knee extended	29.1 (4.7)	0.91	0.83, 0.96	1.41	3.91
		Knee flexed	31.2 (5.1)	0.97	0.97, 0.99	0.88	2.44
Intra-rater reliability of digital inclinometer	Rater 2	Knee extended	30.8 (5.8)	0.95	0.90, 0.98	1.30	3.60
		Knee flexed	31.2 (6.0)	0.91	0.81, 0.96	1.80	4.99
Intra-rater reliability of iPhone level function	Rater 1	Knee extended	30.0 (4.7)	0.85	0.71, 0.93	1.82	5.04
		Knee flexed	30.3 (5.2)	0.95	0.90, 0.98	1.16	3.22
Intra-rater reliability of iPhone level function	Rater 2	Knee extended	27.0 (4.2)	0.85	0.71, 0.93	1.63	4.52
		Knee flexed	30.2 (4.9)	0.95	0.90. 0.98	1.10	3.05

*ICC* intraclass coefficient, *SEM* standard error of measurement, *MDC* Minimal detectable change

**Table 3:** Outcomes of inter-rater reliability of the iPhone level function and the digital inclinometer for the weightbearing lunge test

Measure	Weight bearing lunge position	Mean (SD)	Combined mean (SD)	SEM	ICC	95% confidence interval
Inter-rater reliability of digital inclinometer between raters	Knee extended	*Rater 1* 30.1 (4.6) *Rater 2* 28.1 (4.9)	29.1(4.9)	1.88	0.85	0.59, 0.93
	Knee flexed	*Rater 1* 31.9 (5.1) *Rater 2* 31.0 (5.9)	31.42 (5.5)	1.10	0.96	0.91, 0.98
Inter-rater reliability of iPhone level function between raters	Knee extended	*Rater 1* 27.2 (4.2) *Rater 2* 28.8 (4.5)	27.7 (5.0)	1.22	0.94	0.90, 0.97
	Knee flexed	*Rater 1* 30.2 (4.9) *Rater 2* 31.0 (5.4)	30.4 (4.4)	0.62	0.98	0.96. 0.99

**Fig. 3:**
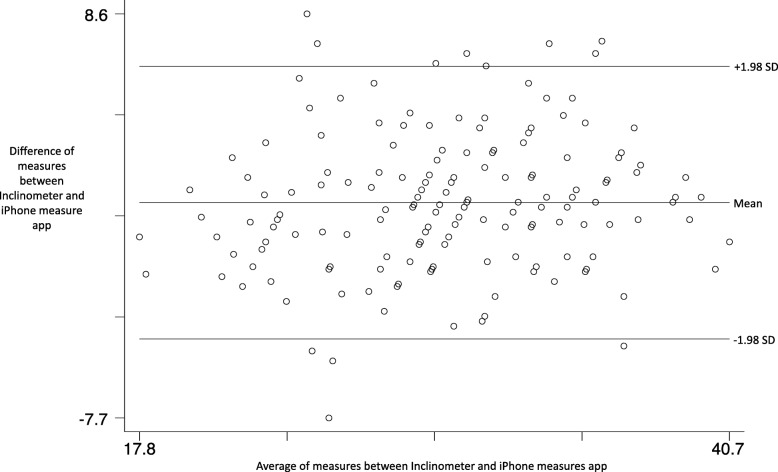
Bland Altman plot of concurrent validity outcomes

Initial concurrent validity, determined between the digital inclinometer and level function on static hard flat and angled (15 degrees) surfaces, was ICC of 1.0 (limits of agreement − 1.0 to 0.61), indicating excellent reliability. There was acceptable concurrent validity between the two devices, and in all leg positions as demonstrated in the Bland Altman plot (Fig. 3). The ICC between all measures, in all positions was good (ICC = 0.84, Mean Difference = 0.99, Limits of agreement = − 4.51 to 6.49) and at least 90% of the plots were within ±1.96 SD (Fig. 3).

## Discussion

To the best of our knowledge this is the first use of the new iPhone level function within the Measure App to review reliability in ankle joint range of motion measures. The outcomes of the study suggest the tool is comparable to digital inclinometers and can be used to measure the weight bearing lunge test in healthy adult populations with confidence.

The weight bearing lunge test with the knee extended and knee flexed has high levels of reliability [7] and is regularly used in research to assess joint range of motion. This includes studies in Charcot Marie Tooth Disease [24], children’s heel pain [6], idiopathic toe walking [25], dancers [17] and changes in plantar pressures in a diabetic population at risk of ulceration [26]. The results from this present study determined intra and inter-rater reliability of all measures were deemed good or excellent. Validity of the level function was also determined as an acceptable comparison to the digital inclinometer, with a low bias and a mean difference close to zero. Within a healthy adult population, the weight bearing lunge test, along with the use of the level function within the iPhone Measure App, can be confidently introduced into clinical practice for quantifying ankle dorsiflexion range of motion.

Similar to previous studies, the knee flexed position demonstrated higher reliability than the straight leg position [13]. The authors proposed that the lower scores with a straight leg may be due to either mechanical placement issues, participant force differences (where potentially more force is placed on the posterior soft tissue structures resulting in increased participant discomfort) or an unknown order effect not dispersed via randomising of participants. With the knee flexed, the measure is presumed to be more of capsular stiffness and less soft tissue impact therefore higher reliability scores were obtained. However, for these reasons the knee extended weight bearing lunge is considered more clinically applicable and is the encouraged measure for research and clinical practice purposes [9, 27, 28].

Whilst these findings encourage clinicians to use the readily accessible technology within their clinic to confidently aid assessment, consideration needs to be given for infection control concerns and phone design. Specifically, mobile phones have previously been shown as an infection hazard [29], the iPhone used within this study did not have a cover and had a flat base. These factors aided positioning but required the phone to be cleaned between and after testing. A cover would not eliminate that cleaning schedule but may alter the flatness of the surface and skin contact. However, these concerns are minimal and can be rectified by following standard cleaning schedules that apply to all other multiple use assessment items used on intact skin.

There are a number of limitations to this study. Experienced raters conducted all measures. Alternative studies on reliability have included a novice rater to compare, therefore care should be taken in considering how these results may apply to the learner user. Additionally, we have suggested that this App is unlikely to change due to its inclusion in the Apple App suite, however, there is still the risk that changes to its function may occur, including but not limited to: the App being removed from the iPhone software; phone case shape variation, or; updates to the Measure App format with changes to the level functionality. Android phone users will need to consider alternative measure Apps as the Measure App is not available on the Android platform. The study was powered with an ICC of 0.6, which indicates moderate reliability [22]. Whilst the research team determined this as an acceptable level, other researchers or clinicians may consider this as low. This should be considered when applying these results in practice or research in the future. Lastly, the mean values of weight bearing lunge were lower than other reported ranges [4, 5], however, comparable to other published values in normative populations [6, 7]. This also highlights that researchers and clinicians should consider the placement of measurement equipment for the weightbearing lunge. Specifically, placement of measurement equipment at tibia’s anterior surface [30] may elicit different results to the equipment’s position as used within this study, and outcomes may not be comparable. It is unknown what impact this may also have on measured reliability.

Future research in the use of this technology for measurement should include understanding the reliability in children and in pathological populations, where there is (potential for) a smaller surface area for device placement. There is also the potential to consider including family/carers in future assessment of this and alternative measuring Apps to determine appropriateness of non-health professional’s ability to determine success where interventions have been prescribed to improve ankle flexibility.

## Conclusion

Using the iPhone level measure, within the Measurement App has demonstrated to be an easy to use and reliable measurement tool for healthy adults. Clinicians should consider how the use of this technology may assist in their clinical practice to assess and measure treatment outcomes.

## Data Availability

Raw data is available from authors and request.

## Abbreviations

App: Application
ICC: intraclass coefficient
SEM: standard error of measurement
